# Histone Deacetylase Inhibitors in Cell Pluripotency, Differentiation, and Reprogramming

**DOI:** 10.1155/2012/184154

**Published:** 2012-03-08

**Authors:** Androniki Kretsovali, Christiana Hadjimichael, Nikolaos Charmpilas

**Affiliations:** ^1^Institute of Molecular Biology and Biotechnology, FORTH, Heraklion, 70013 Crete, Greece; ^2^Department of Biology, University of Crete, Heraklion, 71409 Crete, Greece

## Abstract

Histone deacetylase inhibitors (HDACi) are small molecules that have important and pleiotropic effects on cell homeostasis. Under distinct developmental conditions, they can promote either self-renewal or differentiation of embryonic stem cells. In addition, they can promote directed differentiation of embryonic and tissue-specific stem cells along the neuronal, cardiomyocytic, and hepatic lineages. They have been used to facilitate embryo development following somatic cell nuclear transfer and induced pluripotent stem cell derivation by ectopic expression of pluripotency factors. In the latter method, these molecules not only increase effectiveness, but can also render the induction independent of the oncogenes c-Myc and Klf4. Here we review the molecular pathways that are involved in the functions of HDAC inhibitors on stem cell differentiation and reprogramming of somatic cells into pluripotency. Deciphering the mechanisms of HDAC inhibitor actions is very important to enable their exploitation for efficient and simple tissue regeneration therapies.

## 1. Introduction

Stem cells are distinguished from other cell types by their unique properties to self-renew and differentiate along multiple lineages [1]. These processes are regulated by extrinsic and intrinsic determinants that affect gene expression profiles, signal transduction pathways, and epigenetic mechanisms. 

DNA methylation and histone modifications constitute major mechanisms that are responsible for epigenetic regulation of gene expression during development and differentiation [2–4]. Among other histone modifications, acetylation is very important in nucleosome assembly and chromatin folding. Acetylation favors an open chromatin structure by interfering with the interactions between nucleosomes and releasing the histone tails from the linker DNA. Chromatin regions that are marked by lysine acetylation catalyzed by Histone Acetyl-transferase (HATs) are generally actively transcribed, whereas regions that are bound by Histone Deacetylases (HDACs) bear deacetylated lysines and are inactive [5]. Accordingly, HATs and HDACs reside in multiprotein coactivatory or corepressory complexes, respectively. HATs and HDACs may act either in a site-specific manner, when they are recruited through binding to sequence-specific DNA binding activators or repressors, or in a broad manner whereby they function across large genomic areas.

 There are up to date 18 genes coding for histone (or epsilon lysine) deacetylases in the mammalian genomes. They are grouped in four families. Group I (comprising HDACs 1, 2, 3, and 8). IIa (HDAC 4, 5, 7, 9), IIb (6,10), III (SIRT 1–7), and IV (HDAC 11) [6]. In spite of their name, histone deacetylases have also nonhistone target proteins especially those belonging to group II which do not have histones as substrates. Class I HDACs participate in diverse repressory complexes via interaction with different cofactors such as the Sin3A, Nurd, and CoRest [7]. Contrary to their consideration as repressors, HDACs may act as coactivators of transcription as was reported in the interferon stimulated genes [8]. Genome-wide detection of HATs and HDACs of higher eukaryotic organism has revealed a highly complex situation, active genes are bound by both enzyme types, whereas inactive genes are not bound by HDACs [9]. Inactive genes that were primed for activation by H3K4 methylation were transiently bound by both HATs and HDACs [9].

HDAC inhibitors (HDACis) are natural or synthetic small molecules that can inhibit the activities of HDACs. In spite of similarities in their enzymatic activities, loss of function experiments have attributed highly specific roles to individual members of HDAC proteins in the course of development and differentiation. In addition, HDAC inhibitors that have broad specificity towards their HDAC targets have shown highly specific effects depending on the target cell type [10].

 The profound events that govern stem cell differentiation and somatic cell reprogramming to pluripotency are mainly epigenetic [11]. HDACis are epigenetic modifiers that can promote efficient and temporally regulated control of gene expression. This paper will discuss the role of HDACi in stem cell pluripotency and differentiation as well as in the reprogramming of somatic cells into pluripotency.

## 2. The Role of HDAC Class I and II Members in Mammalian Development and Differentiation

Analysis of knockout mice lacking HDAC genes has revealed their functions during mammalian development and differentiation [10]. HDAC1 gene deletion is embryonic lethal due to cell proliferation and growth defects [12]. The same proliferation defects were reported in HDAC1-null embryonic stem (ES) cells which overexpress the cell cycle inhibitors p21 and p27 [13]. This analysis has revealed a dual role for HDAC1 in both repression and activation of gene transcription. Tissue-specific deletion of HDAC1 in mice did not have significant effect due to functional redundancy with HDAC2 [14]. However, deletion of HDAC2 was reported to cause perinatal lethality in one publication [12], whereas it resulted in a failure to reactivate fetal gene expression programme under cardiac hypertrophic stress in another study [15]. Regarding cardiac growth and development, one allele of either HDAC1 or 2 is sufficient, whereas conditional deletion of both HDAC1 and 2 is lethal due to heart development failure [12].

Similar to the cardiac differentiation, HDAC1 and 2 have essential but redundant roles in the differentiation of neuronal precursors into neurons [12]. Deletion of both enzymes results in severe brain abnormalities and lethality at postnatal day 7 [12]. The roles of individual HDACs 1, 2, and 3 have been assessed in the differentiation of cortical stem cells using dominant negative mutants [16]. Specifically, all three of them inhibit oligodentrocytic differentiation, HDAC2 inhibits astrocytic, whereas HDAC1 is required for neuronal differentiation. On the other hand, specific deletion of both HDAC1 and 2 in oligodendrocyte lineage cells resulted in Wnt pathway activation, which in turn inhibited oligodendrocyte development by repressing Olig2 expression [17]. In agreement with these data, ablation of both HDAC1 and 2 in Schwann cells caused severe myelination deficiency due to NFkB deacetylation [18].

Finally, HDAC1 and 2 have important functions in hemopoiesis [19]. HDAC1 activity is required for erythroid, whereas it blocks myeloid differentiation [20].

HDAC3 deletion is embryonic lethal due to deficient gastrulation [21–23] that is connected to failure in DNA damage repair mechanisms [23]. Conditional tissue-specific deletions of HDAC3 have pointed to an involvement in liver [22] and heart [21] function.

Although class I HDACs are widely expressed, members of the IIa group show tissue-restricted expression. HDAC4 regulates skeletogenesis and knockout mice die in the first week after birth due to excessive ossification of endochondral cartilage which interferes with breathing [24]. This effect is due to unrestricted function of MEF2 and RUNX2, two transcription factors that activate bone formation [25, 26]. RUNX2 is activated by MEF2 and both MEF2 and RUNX2 are targeted by HDAC4 [26]. HDACs 5 and 9 control, in redundant manner, cardiovascular development since single knockout mice are viable, whereas double disruption leads to lethality caused by defective cardiac development resulting from unrestricted activation of MEF2- [27], SRF-, myocardin- and Calmodulin-binding transcriptional activator 2 [28]. In addition, HDAC 4, 5, and 9 control skeletal muscle differentiation through negative regulation of MEF2, PGC1a, and NFAT in response to calcium signals [29] and motor neuron activation [30]. HDAC7 is specifically expressed in endothelial cells of the cardiovascular system [31] and HDAC7 gene deletion results in embryonic lethality due to vascular rupture and excessive hemorrhages [31]. These effects are caused by extreme activation of matrix metalloproteinase (MMP) 10 which is normaly inhibited by HDAC7 [31]. Members of the HDAC class IIb group (HDAC 6, 10) regulate cytoskeletal dynamics by controlling the acetylation of cytoskeletal proteins such as tubulin [32].

HDAC expression and activity are intimately associated with the emergence of neoplasias. In Acute Promyelocytic Leukemia (APL), fusions between Promyelocytic Leukemia (PML) and Retinoic Acid Receptor (RAR) recruit HDACs resulting in the repression of differentiation-related genes [33, 34]. In solid tumors, mutations in HATs [35] and overexpression of HDAC-associated proteins lead to relative hyperactivity of HDAC. Consequently, HDAC inhibitors are long established antitumor agents that were known before the identification of their target HDAC molecules [34, 36].

## 3. Inhibitors of HDACs

HDAC class I and II inhibitors (HDACi) fall into discrete structural categories such as hydroxamic acids, cyclic peptides, benzamides, benzofuranone, and sulfonamide containing molecules [37, 38]. The biological effects of HDACi result from positive or negative regulation of gene expression by induced acetylation of histones, transcription factors or other proteins. Genome-wide analyses of gene expression changes upon HDACi administration have revealed that approximately equal numbers of genes are induced and repressed [39]. The genes affected are highly dependent on the cell type and transformed cells are extremely sensitive as opposed to normal cells. Most studies have been performed with transformed cells. The antitumor activity of HDACi results from a combination of many processes involving cell cycle arrest, apoptosis, activation of mitotic cell death, and inhibition of angiogenesis. In addition, but not unrelated to the aforementioned effects on cell functions, HDACis were reported to induce differentiation of certain cancer cell types [36]. This property gains extreme importance in light of the recently established discovery of “cancer stem cells” [40], a small population of cells that are able to reproduce the tumor and possess self-renewal and pluripotency activities.

## 4. HDAC Inhibitors in Stem Cell Self-Renewal and Differentiation

Due to their activity in epigenetic regulation, HDACis have been widely used in order to alter the differentiation state of stem and somatic cells as shown in Table 1.

### 4.1. Embryonic Stem Cell Pluripotency

Differentiation is a process of gradual loss of potency that ends up to the point where specific cell fate is acquired. Embryonic stem (ES) cells are isolated from the inner cell mass of blastocysts [1, 41, 42] and are characterized by indefinite self-renewal and pluripotency, the capability to follow all potential differentiation pathways [43, 44]. Both mouse and human ES cells express networks of pluripotency transcriptional regulators exemplified by Oct4, Sox2, and Nanog [45]. They differ in the requirements for externally provided cytokines and growth factors. For instance, mouse ES cell culture requires Leukemia Inhibiting Factor (LIF) [46], whereas human ES cell culture depends on Activin/Nodal and FGF [47]. This difference is due to the developmental stages from which these two cell types were isolated. Human ES cells are derived from later stage of embryonic development compared to the mouse and are highly similar to mouse EpiSC (epiblast stem cells) [47–49]. The differentiation of stem cells is very sensitive to epigenetic changes. Therefore, application of epigenetic regulators such as inhibitors of DNA methylation (5 Azacytidine) and HDAC inhibitors may be valuable tools for stem cell interventions [50].

In accordance with their effects on the differentiation of cancer cells, HDACis are able to promote the differentiation of ES cells. Treatment with Trichostatin A (TSA) promotes morphological and gene expression changes reminiscent of differentiation even in the presence of LIF [51, 52]. Inhibition of HDAC activity accelerated the early differentiation steps of ES cells without being sufficient for commitment to a specific lineage. Genome-wide analysis revealed two gene groups that are targeted by TSA: the first one contains genes related to pluripotency that are suppressed by TSA (Sall4, Nanog, Klf4, Oct4, and Sox2), the second is required for lineage-specific differentiation and its expression is upregulated by TSA [52].

In contrast to these studies, other studies have shown that HDACis increase self-renewal and interfere with differentiation. Low doses of TSA (10 nM) reverted mouse embryoid bodies towards the undifferentiated state [53] and employment of sodium butyrate (NaBu) was reported to support human and mouse ES cells self-renewal when administered within a narrow range of concentrations [54]. In the latter study, low doses of butyrate (and TSA) were able to substitute for FGF2 (human ES) and LIF (mouse ES). However, higher doses led to differentiation. Surprisingly, nonoverlapping transcriptional expression profile changes were observed in butyrate-treated human and mouse ES cells [54]. These findings have shown the ability of butyrate to modulate the stem cell stage pushing mouse ES forward and pulling human ES backward [54]. In agreement with these data, treatment of mouse ES with TSA was able to shift a population of epiblast-like ESC towards an ICM-like state [54, 55]. A conclusion of all these studies might be that HDACis exert an antidifferentiation effect when low doses are applied on cells that have already exited from self-renewal either as embryoid bodies [53] or epiblast-like [54, 55] cells, whereas higher doses applied on undifferentiated cells provoke differentiations [51, 52]. The same effect was observed upon HDACi treatment of two embryonic carcinoma (EC) cell lines F9 and P19. In F9 cells which belong to a less differentiated state, the expression of the pluripotency factor *Fgf4* decreased after treatment with Valproic acid (VPA) and TSA. In contrast, the same treatment of P19 cells, which are more differentiated, caused the elevation of *Fgf4* expression [56]. In agreement with this data, reactivation of pluripotency genes such as Oct4, Nanog, and Klf4 was observed in neurosphere cells treated with TSA and azacytidine, AzaC [57]. Hence, changes in the acetylation levels of stem cells result in alterations of the differentiation status in correlation with the developmental stage.

Directed differentiation of ES cells is not easy to control. Differentiation protocols generally rely either on the generation of ES cell aggregates (embryoid bodies) or on culturing on stromal cells. Effectiveness and selectivity need to be significantly improved in order for ES cell to be used as tools for cell-based therapies.

HDACi treatment was used for directed differentiation of mouse ES cells towards the cardiomyocytic lineage. TSA added on embryoid bodies between days 7 and 8 potentiated cardiac differentiation due to hyperacetylation of GATA4 [58] a master regulator of cardiogenesis. In addition, TSA induced, whereas HDAC4 overexpression inhibited, cardiomyogenesis of embryonic carcinoma P19 cells [59]. TSA was also able to facilitate the myocardial differentiation of induced pluripotent stem cells [60]. Interestingly, TSA and NaBu were reported to induce HDAC4 proteasomal degradation which in turn results in MEF2 activation and cardiac lineage commitment [61]. On the other hand, NaBu was proven effective in the induction of pancreatic and hepatic differentiation from mouse and human ES cells [62–64].

### 4.2. Tissue-Specific Stem Cells

#### 4.2.1. Neural Stem Cell Differentiation

As indicated previously ablation of HDAC1 and 2 is postnatal lethal due to disorganization of brain structures [12]. However, administration of HDAC inhibitors led to the induction of neuronal and suppression of glial differentiation [65]. In addition HDAC activity is required for timing of oligodendrocyte differentiation [66].

Specifically, VPA was reported to increase neuronal differentiation of adult neural progenitor cells and inhibit astrocyte and oligodendrocyte differentiation [65]. Moreover, VPA administration inhibited the differentiation of oligodendrocyte progenitor cells in the developing rat brain [66].

The molecular mechanism of VPA function was induction of NeuroD, a neurogenic bHLH transcription factor [65]. Derepression of NeuroD and neuronal fate activation was also caused by HDAC5 nuclear exclusion [77]. In another study, VPA promoted neuronal fate commitment via activation of the ERK pathway [70]. TSA was able to increase differentiation of neural stem cells at the expense of astrocyte production [82]. Importantly, the TSA-produced nerve cells bear normal electrophysiological properties and morphological characteristics such as the extension of long dendrites with branching points. Treatment of Adult Subventricular Zone (SVZ) precursor cells with MS-275, M344, and suberoylanilide hydroxamic acid (SAHA) increased neuronal differentiation and inhibited oligodendrocyte production via induction of NeuroD cyclinD2 and B-lymphocyte translocation gene 3 [84]. VPA was reported to promote neuronal differentiation of hippocampal neural progenitor cells by induction of proneural factors Ngn1, Mash1, and p15 and histone H4 acetylation [85]. Combination of TSA with Shh, Fgf8, and Wnt1 promotes differentiation of nonmecencephalic neural stem cells to dopaminergic neurons [69]. Interestingly, the regulatory role of histone acetylation in the nervous system is evolutionarily conserved between vertebrates and invertebrates. High levels of acetylation are required for neuronal, whereas low levels are connected to the glial differentiation of Drosophila neural stem cells [86].

#### 4.2.2. Hemopoietic Stem Cell Self-Renewal and Differentiation

Mouse and human hemopoietic stem cells (HSC) self-renewal was potentiated by chlamydocin [87]. In another study, the application of TSA with 5-AzaC increased 12.5-fold the proliferation of HSC isolated from umbilical cord HDACi [73, 74].

Mesenchymal stem cells (MSCs) from adipose tissue and umbilical cord blood were treated with two HDAC inhibitors, VPA, and NaBu [88]. Posttreatment controlled differentiation was conducted into bone, fat, cartilage, and nervous tissue. Different results were obtained depending on the cell types which were examined. VPA and NaBu attenuated the efficiency of adipogenic, chondrogenic, and neurogenic derivation. On the other hand, osteogenic differentiation was elevated after HDACi treatment. An interesting new prospect has arisen following a publication which supports that HDAC inhibitors can be used to direct pancreatic cells to a specific lineage. It was shown that NaBu and TSA promote ductal differentiation at the expense of the acinar fate [78]. Thus, cells with exocrine function are converted to endocrine cells, capable of producing hormones such as insulin and somatostatin [89].

#### 4.2.3. Cardiomyocytic Differentiation

Cardiac side population cells isolated from rat hearts were coaxed in cardiomyocytic differentiation by TSA treatment [90]. TSA induced the expression of transcription factors Nkx2.5, GATA4, and MEF2C that play important roles in the orchestration of the events that lead to the production of cardiomyocytes, endothelial, and smooth muscle cells [90]. In another study, TSA and azacytidine treatment promoted cardiomyocytic differentiation of mesenchymal stem cells via induction of the same transcription factors GATA-4, NKx2.5, and MEF2c [91].

## 5. HDAC Inhibitors in Cell Reprogramming to Pluripotency

Reprogramming differentiated somatic cells to pluripotent stem cells has emerged as a way of producing patient-specific stem cells. These cells can be possible candidates for regenerative medicine after their differentiation to a specific cell fate.

A strategy used to reverse the differentiated state of cells was somatic cell nucleus transfer (SCNT) to enucleated eggs or oocytes [92, 93]. This proved in an emphatic way the fact that cell differentiation is not an irreversible process and that the nucleus of a differentiated cell can be reprogrammed to follow a dedifferentiation program. Additionally, it is a common belief that the more ancestral a cell is, the easier it is to be reprogrammed using the method of nuclear transfer. There are several reports showing that HDAC inhibitors can in fact be very helpful tools in the attempt to increase the efficiency of nuclear transfer experiments (Table 1).

Early reports have applied TSA to donor cells [94] or to the embryos following SCNT [68, 72] and shown that it improves both the in vivo and in vitro developmental rate. TSA was effective as cloning facilitating reagent for many species embryos, bovine ([95], mouse ([71, 96]), and porcine ([97]). TSA treatment caused chromatin rearrangements such as elevated histone acetylation and chromosome decondensation as well as increased rate of RNA synthesis [98]). Treatment of SCNT-generated mouse embryos with scriptaid improved the cloning efficiency for various inbred strains [96]. Moreover, scriptaid treatment resulted in higher levels of nascent mRNA transcription at the two-cell stage and this increase depended on the genotype of the mouse strain used. The cloned mice were both viable and fertile and there was a positive correlation between the increase in nascent mRNA synthesis and full-term development of cloned mice [96]. A different HDACi, CBHA, was reported to augment the developmental potential of cloned mouse embryos at both the pre- and postimplantation stages. Furthermore, CBHA treatment resulted in a statistically significant increase in the total ICM cell number, simultaneously reducing the ratio of apoptotic cells. In addition, it was shown that Oct4 expression was more abundant in the population of cells isolated from blastocysts of treated animals than untreated ones. Hence, those cells resembled ES cells as was confirmed by staining for pluripotency markers (Sox2, SSEA1, alkaline phosphatase) [71]. Finally two other HDAC I and IIa/b inhibitors suberoylanilide hydroxamic acid (SAHA) and oxamflatin could improve the development of cloned mice by reducing the apoptosis in blastocysts [99].

In a pioneer work, the group of Yamanaka [100] reprogrammed fetal and adult mouse fibroblasts to induced Pluripotent Stem (iPS) cells using four key transcription factors, namely Oct4, Sox2, c-Myc, and Klf4. A year later human fibroblasts were reprogrammed by the group of Takahasi et al. [101] and Park et al. [79], whereas the group of Thomson substituted the oncogenic factors Klf4 and c-Myc with Nanog and Lin28 [102]. The aforementioned iPS cells possess identical characteristics with ES cells, such as expression of pluripotency markers, ES cell morphology, self-renewal, and capability of teratoma formation [79, 101].

In order to improve the efficiency of reprogramming, several strategies were developed [83] using small molecules such as DNA methyltransferase inhibitors (5 AzaC, [75]), histone methylotransferase inhibitors (BIX, [76]), and HDAC inhibitors (Table 1). Important steps have been made towards the direction of replacing the oncogenic factors with chemical compounds. In particular, Valproic acid (VPA) was used to substitute for c-myc [80]. VPA and the pluripotency factors Oct4, Sox2, and Klf4 were able to reprogram primary human fibroblasts. The presence of VPA increased the number of iPS colonies by 50-fold. Produced iPS cells resemble ES cells in pluripotency and gene expression profiles [80]. In another study, Klf4 was fully dispensable [67]. The combination of Oct4, Sox2, and VPA was sufficient to reprogram somatic cells with a similar efficiency compared to three-factor reprogramming (Oct4, Sox2, and Klf4). These iPS cells exhibited several desired characteristics, such as increased levels of pluripotency markers and alkaline phosphatase activity. In addition, they seemed morphologically similar to human ES cells and were karyotypically normal. Finally, the two factor-induced human iPS cells were able to form teratomas derived from all three lineages. It is possible that VPA treatment sets somatic cells in a transition state before their complete dedifferentiation [67]. These results offer great possibilities in attaining full reprogramming with chemical reagents, a procedure both safe and practical to be used in human therapies.

In a recent study [75], human fetal fibroblasts were reprogrammed to pluripotency using human ES cell extracts with the addition of 5-azacytidine, TSA, and retinoic acid. This proves that the epigenetic state of cells has a great impact on the efficiency of reprogramming by this method. During the process, upregulation of pluripotency markers (Oct4, Sox2) and morphological changes were observed. In parallel, markers of differentiation (LAMIN A/C) were downregulated, showing a positive correlation between dedifferentiation, and increase in acetylation status of cells.

Another HDAC inhibitor NaBu used at low doses improved the generation of iPS cells by 50-fold by using retroviral or “piggyback” vectors for reprogramming human fibroblasts even in the absence of Klf4 and c-myc [81]. In another study, butyrate was reported to potentiate iPS cell generation from mouse embryonic fibroblasts in the presence of c-myc [103]. This difference might be due to differences in the endogenous c-myc levels between the human and mouse cells.

In addition to the typical iPS cells, reversion of differentiation was assisted by the addition of HDACi in other cell types. Dedifferentiation of primordial germ cells (PGC) into pluripotent embryonic germ (EG) cells was achieved using TSA to replace FGF-2 [104]. A high-throughput screen has revealed the ability of four HDAC inhibitors (NaBu, TSA, MS-275 and Apicidin) to reprogram oligodendrocyte progenitors (OPC) into multipotent neural stem-like cells that can generate both neurons and glia [105]. Finally, an intriguing new possibility emerged from a recent publication using the nematode *C. elegans* as model [106]. The researchers employed two common HDAC inhibitors (VPA and TSA) to mimic the removal of histone chaperone LIN-53 and managed to reprogram germ cells into specific neuron types. It would be interesting to examine the effect of HDAC inhibition in efforts of direct reprogramming from one type to the other in the more complex context of mammalian cells.

## 6. Conclusions and Perspectives

Stem cell methodologies have revolutionized modern therapeutic strategies that aim to replace damaged cells or tissues. Controlling the pluripotent stem cell fate [95] is dependent on important transcription, signaling, and epigenetic factors. Among other epigenetic regulators, Histone deacetylases have important roles in cell physiology, differentiation, developmental decisions, and tumor formation [10]. Compared to HDAC genes deletions, HDAC inhibitors elicit cell restricted, albeit pleiotropic effects. A vast collection of natural and synthetic HDAC inhibitors has shown very potent effects in embryonic stem cell differentiation pathways. They may promote either self-renewal [54, 55] or differentiation [51, 52] depending on the stem cell status and the dose employed. These effects might result from reorganization of the embryonic stem cell chromatin that is remarkably dynamic and decondensed [107]. Therefore, HDACi can reverse the repressive or activating epigenetic traits that characterize genes involved in the regulation of self-renewal or differentiation.

Most importantly, HDACis have shown considerable activity in directing the neuronal, cardiomyocytic, and hepatic lineages differentiations. In most cases where the molecular mechanism was examined, it involved the induction of differentiation-regulating transcription factors. Moreover, HDACis were used in somatic cell reprogramming processes. Treatment of donor cells before transfer or embryos following transfer resulted in facilitation of embryo cloning and improvement of embryo developmental potential. These effects were due to enhanced histone acetylation, chromatin decompaction, increase of RNA synthesis, and inhibition of apoptosis. Due to the ethical issues raised by embryo cloning, these techniques are not yet applicable to humans. Therefore, the recent achievement of iPS generation has offered great expectations in custom-specific stem cells for human health. In that field, there is increasing effort in omitting retroviral vectors, oncogenes, and—if possible—all kinds of exogenous genetic material. Substituting transcription or signaling factors with simple small molecule reagents can render the therapies both safer and simpler. For that purpose, HDAC inhibitors have shown activity to enhance reprogramming and substitute for the presence of transcription factors, importantly the oncogenes c-myc and Klf4 [67]. However, the exact molecular mechanism whereby VPA, TSA, and other HDACi function needs to be elucidated. Future researches are expected to elucidate the mechanism of HDACi action in order to design novel reagents with increased effectiveness and specificity. On the other hand, genome-wide analyses have shown that acetylation is a modification as frequent as phosphorylation. Considering that nonhistone proteins are also targets for acetylation, it is expected that analysis of the “acetylome” [108, 109] changes in the course of stem cell differentiation will shed light on the functions and applications of HDAC inhibitors. In addition to mRNA profiling, analysis of miRNA expression changes that follow HDACi may reveal mechanisms whereby these reagents have so specific effects on different cell differentiation backgrounds. HDACis are able to potentiate both stem cell differentiation and somatic cell reprogramming to pluripotency. This may suggest that common mechanisms are involved in opposite changes of the differentiation status. Elucidation of these mechanisms is expected to open new opportunities in the interface between chemistry and stem cell biology. Combining HDAC inhibitors with other small molecule effectors and miRNAs [110] can provide valuable tools to overcome challenges due to genetic interventions and improve stem cell applications for tissue regeneration therapies.

## Figures and Tables

**Table 1:** Functions of HDAC inhibitors in stem cell self-renewal or differentiation and somatic cell reprogramming to pluripotency.

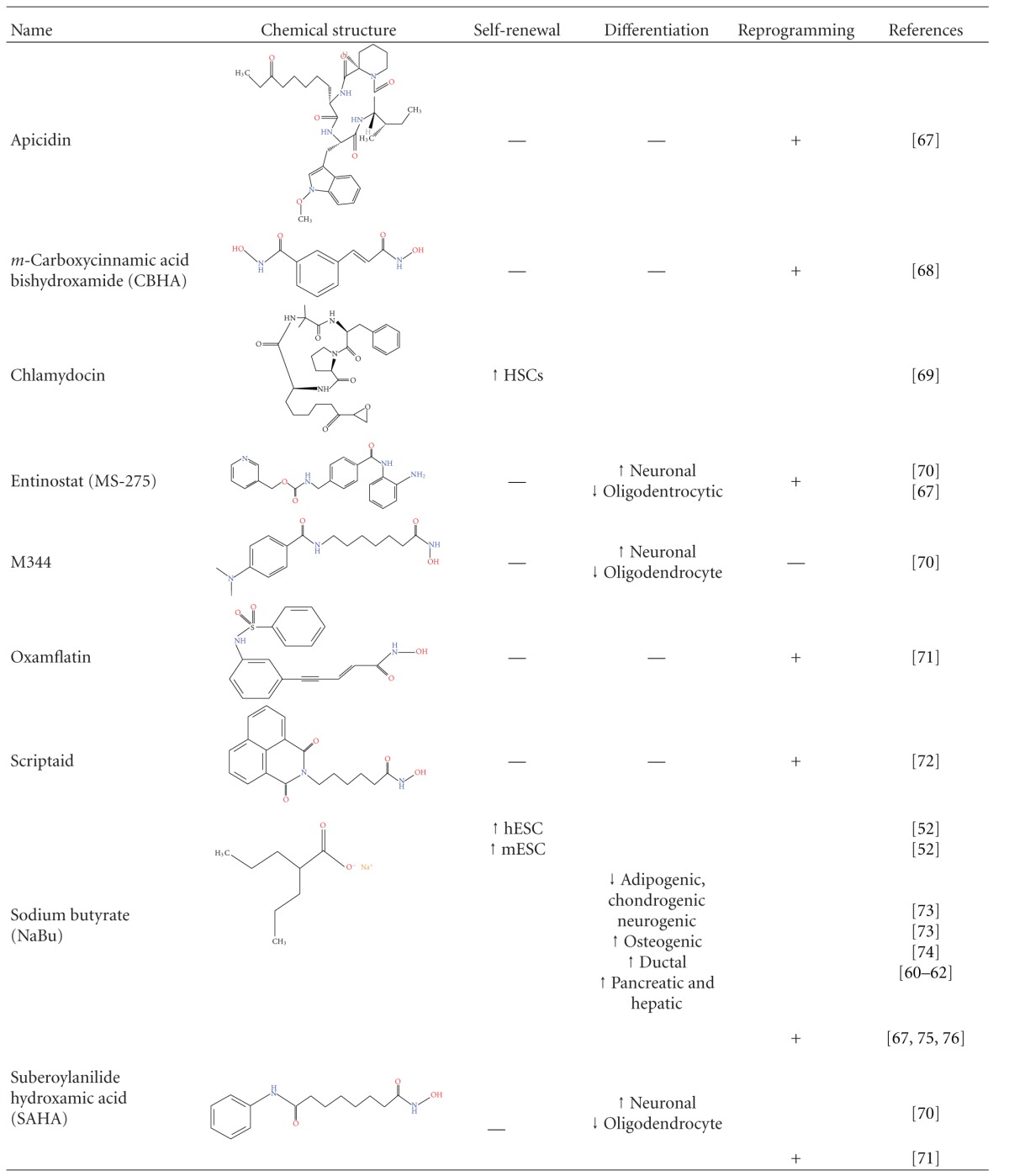 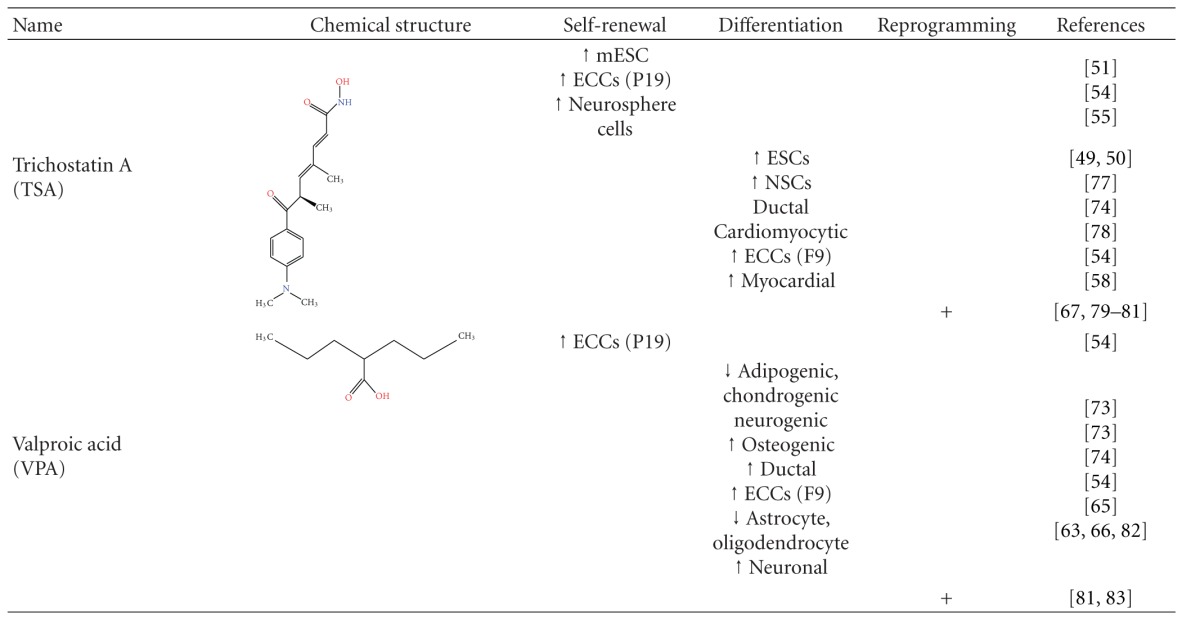

Embryonic Stem Cells (ESCs), embryonic carcinoma Cells (ECCs), hemopoietic stem cells (HSCs), and neural stem cells (NSCs).
